# Evolving Treatment Paradigm in the Management of Diabetic Macular Edema in the Era of COVID-19

**DOI:** 10.3389/fphar.2021.670468

**Published:** 2021-04-12

**Authors:** Claudio Iovino, Enrico Peiretti, Giuseppe Giannaccare, Vincenzo Scorcia, Adriano Carnevali

**Affiliations:** ^1^ Multidisciplinary Department of Medical, Surgical and Dental Sciences, University of Campania Luigi Vanvitelli, Naples, Italy; ^2^ Department of Surgical Sciences, Eye Clinic, University of Cagliari, Cagliari, Italy; ^3^ Department of Ophthalmology, University of Magna Graecia, Catanzaro, Italy

**Keywords:** COVID-19, intravitreal injection, diabetic macular edema, dexamethason implant, pandemic (COVID19)

## 

Intravitreal therapy is widely recognized as a major milestone in ophthalmology being one of the most commonly performed ocular procedures (He et al., 2018). The spread of coronavirus disease (COVID-19) still represents an important public health problem worldwide (Ferrara et al., 2020; Wang et al., 2020). This novel virus infection, is causing a significant downsizing of non-urgent treatments provided for ocular disorders (Tognetto et al., 2020; Toro M. D. et al., 2020, Toro M. et al., 2020), including intravitreal therapy (Elfalah et al., 2021).

Since diabetic retinopathy (DR) still remains the leading cause of blindness among working-age adults (Ting et al., 2016), ophthalmologists should be aware of the potential negative effects of COVID-19 restrictions in the management of diabetic patients in the next months.

The global COVID-19 pandemic led many governments from different nations to adopt protective and strict measures to reduce its spread. In these unprecedented circumstances, many healthcare systems are overwhelmed and under stress.

In this scenario, there is an urgent need to support ophthalmologists who are treating patients with intravitreal injections in decision-making protocols. In order to provide continuity of care, and to reduce the risk of contamination, series of protection measures have been proposed (Iovino et al., 2020a; Borrelli et al., 2020; Korobelnik et al., 2020). Nevertheless, many patients cannot receive a prompt therapy due to all public health restriction measures. During COVID-19 outbreak Carnevali et al. proposed treatment priority levels to treat the most urgent patients, although a drop of 91.7% of the injections performed compared to the same period of 2019 was registered (Carnevali et al., 2020).

Diabetic patients are considered at high risk for COVID-19 complications and should not be exposed to avoidable risks, including the injections procedure itself. However, continuation of care, where possible, is important to avoid irreversible vision loss.

For non-monocular patients with diabetic macular edema (DME), postponement (>4–6 months) of appointments has been proposed (Korobelnik et al., 2020). As recently reported, postponing treatment in patients with good visual acuity does not affect the prognosis at 1 year, regardless of whether the DME was treated or not (Busch et al., 2019). Conversely, in patients with more advanced DR and worse visual acuity, a delay in treatments could cause irreversible visual loss (Ting et al., 2016; Elfalah et al., 2021).

Anti-vascular endothelial growth factor (VEGF) injections represent generally a first-line therapy for several retinal disorders including DME (Heier et al., 2012; Reibaldi et al., 2014; Schmidt-Erfurth et al., 2017; Plyukhova et al., 2020), but monthly injections are needed at least during the loading dose (Schmidt-Erfurth et al., 2017). Of note, intravitreal dexamethasone (DEX) implant 0.7 mg (Ozurdex®, Allergan, Inc. Irvine, CA, United States) is considered a valid alternative for both refractory to anti-VEGF treatment eyes and treatment naïve ones (Iglicki et al., 2019; Iovino et al., 2020b). Intravitreal DEX implant releases active ingredients within the vitreous chamber over a 3–6 months period, and its efficacy and safety in various retinal diseases have been proved in clinical trials and real-life studies (Maturi et al., 2016; Rajesh et al., 2020). Several authors also reported significant anatomical and functional effects of DEX implant in vitrectomized eyes in different conditions (Boyer et al., 2011; Reibaldi et al., 2012; Iovino et al., 2019). Corticosteroids have multiple levels of action, modifying tight junction integrity, inhibiting different molecules involved in vascular permeability and inflammation processes including interleukin-6, stroma-derived factor-1, Intercellular adhesion molecule-1, as well as VEGF (Iovino et al., 2020b).

All these mechanisms of action work in aggregate, resulting in decreased macular edema and VEGF production, fibrin deposition, capillary leakage and migration of inflammatory cells (Gagliano et al., 2015). There is evidence that oxidative stress, ischemia and inflammation promote the initiation and progression of DR (Toro et al., 2019), further supporting the role of DEX implant in controlling the progression of the DME (Ceravolo et al., 2020).

Cataract progression and intraocular pressure rise are the most common side effects, but often rather easily manageable (Iovino et al., 2020b; Rajesh et al., 2020). Additionally, several optical coherence tomography (OCT) biomarkers were identified as functional outcome predictors in DME eyes treated with DEX implant including the presence of submacular fluid, absence of hyperreflective intraretinal foci and integrity of the ellipsoid zone (Zur et al., 2018).

On this background, a good selection of patients with DME who can benefit from observation or a single intravitreal DEX injection rather than monthly anti-VEGF injections, could be of great importance in reducing the burden of injections of clinics and hospitals. Treating eligible subjects with DME showing the previously mentioned OCT biomarkers, could indeed reduce the burden of care delivery for patients and health system. Considering that the IOP increase after the injection is typically noticed within the first 2 weeks, IOP lowering eye drops together with a post-injection visit should be taken into account for patients with high risk for glaucoma.

Almost one year is gone since the WHO declared the global pandemic and new more contagious virus variants are now emerging. Physicians may be dealing with this emergency status for the next 1 or 2 years.

In our opinion, by tailoring the treatment to patients in most need, equity can be considered the ethical value that support the decisionmaking by the treating provider.

Although an evidence-based clinical practice guideline for intravitreal injections is not yet available, we believe that these considerations about management of diabetic patients with DME, could be useful for ophthalmologists from most affected countries who will be under public health COVID-19 measures and restrictions for the next months. Saving costs, resources and time is an important goal for all health workers who are facing this common enemy in first line.
